# Oscillatory brain responses to emotional stimuli are effects related to events rather than states

**DOI:** 10.3389/fnhum.2022.868549

**Published:** 2023-01-18

**Authors:** Lisa Luther, Jörn M. Horschig, Jacobien M. van Peer, Karin Roelofs, Ole Jensen, Muriel A. Hagenaars

**Affiliations:** ^1^Behavioural Science Institute (BSI), Radboud University, Nijmegen, Netherlands; ^2^Donders Institute for Brain, Cognition and Behaviour, Radboud University, Nijmegen, Netherlands; ^3^School of Psychology, Centre for Human Brain Health, University of Birmingham, Birmingham, United Kingdom; ^4^Department of Clinical Psychology, Utrecht University, Utrecht, Netherlands

**Keywords:** IAPS, EEG, alpha band activity, gamma band activity, stimulus induced, state effect, balance board, body sway

## Abstract

Emotional cues draw attention, thereby enabling enhanced processing. Electrophysiological brain research in humans suggests that increased gamma band activity and decreased alpha band activity over posterior brain areas is associated with the allocation of attention. However, emotional events can alternate quickly, like rapidly changing news items and it remains unknown whether the modulation of brain oscillations happens in a stimulus induced manner, changing with each individual stimulus, or whether the events lead to prolonged, state-like changes. To investigate this, we measured the electroencephalogram (EEG) during a passive viewing task (*N* = 32) while emotional pictures International Affective Picture System (IAPS) were presented in blocks containing either *pleasant* and *neutral* or *unpleasant* and *neutral* pictures. As predicted, we found decreased alpha and increased gamma power over posterior areas in response to *unpleasant* compared to *pleasant* pictures (and also compared to *neutral* pictures for gamma power). When testing the *neutral* pictures of the unpleasant and pleasant block against each other, we found no significant difference, which speaks to a stimulus induced effect of alpha and gamma power rather than a state effect. In addition, the inter-trial interval (ITI) between the pictures did not differ between the unpleasant and pleasant block either, corroborating this conclusion. Since emotional pictures can at the same time elicit a freezing-like response and we were interested in whether this freezing-like response co-occurs with enhanced attention, we also collected postural sway data. However, within this EEG-setup, postural analyses indicated no stimulus-related effects nor a correlation with EEG-data. We interpret the alpha and gamma band results as reflecting event-related attention toward unpleasant compared to pleasant (and neutral) pictures and discuss this finding in light of previous EEG research and in combination with behavioral research on threat-induced reductions in body sway (freezing-like response).

## Introduction

Emotional content is a strong determinant of the degree of attention that we direct toward a given visual object. Brain oscillations are known to be modulated in different frequency bands as a consequence of visual gating as for instance modulated by selective attention (Klimesch et al., 2007; Jensen and Mazaheri, 2010; Foxe and Snyder, 2011). The alpha rhythm (8–14 Hz) has been shown to reflect inhibition of task-irrelevant brain areas during the allocation of spatial attention (Klimesch et al., 2007; Haegens et al., 2012; Okazaki et al., 2015; Zazio et al., 2020). While this relation between alpha band activity and attention is known for spatial attention tasks, we set out to investigate how alpha band activity as a read-out measure of attention is modulated by the emotional valence of pictures. In several recent studies using electroencephalogram (EEG) it was shown that alpha power decreases more for emotional compared to less salient stimuli (Keil et al., 2001; Balconi et al., 2009; Popov et al., 2012). Keil et al. (2001) demonstrated a stronger decrease of alpha power (i.e., alpha suppression) for unpleasant compared to both pleasant and neutral pictures during right hemifield presentations. Interestingly, they found the opposite effect for left hemifield presentations (a stronger alpha power decrease for pleasant pictures). Popov et al. (2012) also found posterior alpha suppression for unpleasant compared to neutral pictures. However, Balconi et al. (2015) did not find effects of either valence or arousal of affective pictures on alpha power in a passive viewing task.

Besides alpha activity, gamma band activity has been shown to reflect active processing and to increase with attention (Fries et al., 2001). Furthermore, there is evidence for increased gamma activity in response to emotional compared to neutral stimuli, albeit the reported frequency band of gamma varies across the studies. Balconi et al. (2009) found wide-spread increased gamma power (40–50 Hz) in response to highly arousing pictures (pleasant and unpleasant) compared to low arousing pictures. Keil et al. (2001) also found 45–65 Hz gamma power increases over the right hemisphere for arousing (unpleasant and pleasant) compared to neutral pictures. Aftanas et al. (2004) found a similar effect over parieto-occipital areas in the 30–45 Hz band. Mueller et al. (1999) reported increased gamma power (30–50 Hz) over right frontal and temporal sites for arousing compared to neutral pictures, but no effect was observed for gamma power between 50 and 90 Hz. In a block design, Güntekin and Tülay (2014) found higher gamma power in response to unpleasant pictures compared to pleasant and neutral pictures over occipital electrodes.

In sum, previous studies showed modulation of alpha and gamma power by emotional stimuli, although the results for alpha power are somewhat inconsistent. However, all of these studies used statistical approaches based on pre-defined time windows, frequency ranges, and/or topographical sites. We set out to investigate the power modulations during emotional picture viewing by using a non-parametric statistical test, free from *a priori* defined time periods and frequencies (Maris and Oostenveld, 2007) for two reasons. First, this test assures an adequate statistical sensitivity to detect condition differences. Second, by not pre-defining the frequency and time ranges, and including all channels in the analysis, we allow for bottom-up discovery of effects in terms of frequency range and time window, and the topographical pattern of activity will reveal in a bottom-up manner the sites that form part of the largest cluster of activation difference due to condition. Using such an approach, Popov et al. (2012) found gamma power increases (60–80 Hz) for unpleasant compared to neutral pictures; however, they did not have arousing pictures of pleasant valence in their design.

The encounter of aversive information such as unpleasant pictures not only affects attention, but has also been shown to elicit freezing-like responses in humans (Lang et al., 1997; Hagenaars et al., 2012, 2014), i.e., a state of immobility in the face of threat. This can be detected by measuring postural movement (body sway) using a stabilometric platform (Azevedo et al., 2005; Facchinetti et al., 2006; Stins and Beek, 2007; Hagenaars et al., 2012; Niermann et al., 2015) and is usually found in a state-like fashion (i.e., on block-level). Animal models posit that freezing is a state of enhanced attention (Lang et al., 1997), but the link between allocation of attention and freezing is unknown in humans. Therefore, the brain oscillations associated with freezing deserve further investigation. In particular, we were interested if the power modulations by emotional pictures, as described above, reflect the immediate (i.e., stimulus induced) modulation of attention or whether longer lasting (i.e., state-like) effects of emotion also contribute to this attention process, as was found for body sway.

With these considerations in mind, we conducted an EEG study in which participants were shown pictures of unpleasant, pleasant and neutral valence while standing on a stabilometric platform (balance board). We explored whether these oscillatory modulations are state-like (as freezing is a state-like response) or stimulus induced (as most EEG research on emotional pictures finds stimulus induced effects). Further, we hypothesized that in particular unpleasant pictures would elicit a stronger alpha decrease and gamma power increase compared to the other pictures. Lastly, we posited that these oscillatory modulations would relate to freezing (a reduction in body sway for unpleasant compared to pleasant and neutral pictures), i.e., that people who froze more would also show a stronger alpha power decrease and gamma power increase toward unpleasant pictures as compared to pleasant and neutral pictures.

## Materials and methods

### Participants

Forty-four healthy volunteers (18 male) were recruited from Radboud University Nijmegen, *via* an experiment participation system, flyers, and personal contacts. Due to technical problems leading to incomplete data sets, data from 4 participants were excluded for the EEG analysis, and 7 for the analysis of stabilometric data (with an overlap in participants). Additionally, 3 more participants dropped out during the course of the experiment due to dizziness or feeling unwell. The mean age of the 34 remaining participants (12 male) was 24.16 (*SD* = 7.16). The study was approved by the local ethics committee (Ethische Commissie Gedragswetenschappelijk onderzoek, ECG) and participants gave written informed consent prior to the experiment.

### Materials

#### Stimuli

Each participant was presented 208 pictures from the International Affective Picture System (IAPS; Lang et al., 2001) of which 52 were pleasant, 52 unpleasant, and 104 neutral pictures (see Appendix). Pictures were selected based on the normative valence ratings provided by the IAPS manual (cut-off values: unpleasant <3.2; neutral: 4–6; pleasant >6.8). The valence ratings were further constrained not to differ more than 0.8 points between men’s and women’s ratings. The pictures were matched in terms of luminance and size with a custom written program (MatLab script). Each category included the same ratio of pictures including vs. excluding humans (including the face).

Each picture appeared only once during the passive viewing task. Pictures were presented in 8 blocks consisting of 26 pictures. Each block contained 13 neutral pictures and 13 emotional (either pleasant or unpleasant) pictures. This amounted to 4 blocks of pleasant and neutral pictures, and 4 blocks of unpleasant and neutral pictures. This arrangement lead to four conditions on the picture level: (1) pleasant, (2) neutral-in-pleasant-block, (3) unpleasant, and (4) neutral-in-unpleasant-block.

### Procedure and experimental paradigm

**FIGURE 1:**
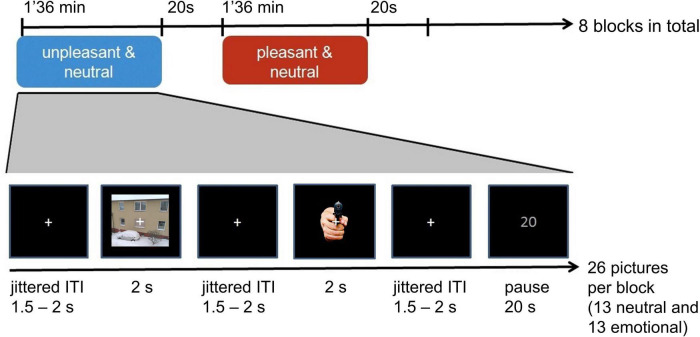
Experimental paradigm. Passive viewing task during which participants were gazing at the fixation cross while perceiving the picture around the cross. Neutral pictures were presented among either pleasant or unpleasant pictures.

Participants took part in a larger study, of which the third part consisted of the passive viewing task of the IAPS pictures, the data of which are presented here. The first part consisted of the same study design as described here (passive viewing task) but with emotional *faces* and the second part of a memory task on those faces. Recordings took place in a specialized EEG lab which was quiet, without daylight and undisturbed. Participants were standing on a stabilometric force platform while the EEG was simultaneously recorded. Stimuli were presented on a Samsung 2233SW screen with a refresh-rate of 60 or 120 Hz differing between subjects, which was adjusted to eye-height. The refresh rate differed due to a change in the technical circumstances in the laboratory but did not show in the data nor affect stimulus presentation times. The pictures and blocks were pseudo-randomized being constrained to not more than two blocks of one valence (pleasant or unpleasant) sequentially, and within each block not more than four pictures of one valence (neutral or pleasant, or neutral or unpleasant, respectively) sequentially. Pictures were presented for 2 s with inter-trial intervals (ITIs) of 1.5–2 s, leading to a total duration of around 1 min 36 s per block. The blocks were separated by 20 s breaks. After four blocks followed a 3 min break allowing participants to sit down and rest their legs. This design represents the best current combination between EEG and balance board designs, compromising between the requirements of each method (i.e., block designs used in balance board studies and event-related designs as used in EEG studies). A white fixation cross stayed on the screen and the pictures were presented on top (Figure 1). During the break this cross turned into a countdown to give the participants an orientation as to how long they still had time to relax and move their eyes before resuming the picture presentation.

Participants were asked to fixate on the cross while perceiving the picture around it. Furthermore, they were asked to blink after each picture in order to reduce blinking during stimulus presentation. Their posture was required to be relaxed with arms hanging alongside the body.

### Data acquisition

#### Electroencephalography

A 64 channel EEG system (ActiCap64 system, Brain Products GmbH, Gilching, Germany) based on the extended 10-10 system was applied. The signals were referenced to linked mastoids and later re-referenced to a common average reference. The data were sampled at 500 Hz following band-pass filtering between 0.016 and 150 Hz.

#### Stabilometric platform

The recordings of the body sway took place on a custom build stabilometric square platform (50 × 50 cm; 19.7 inches) with a force sensor in each corner (distance between sensors: 42.5 cm; Niermann et al., 2015). The stabilometric data were acquired together with the EEG using four additional E × G channels.

### Data analysis

#### Electroencephalography sensor level analyses

Electroencephalography data were analyzed using MatLab and the MatLab-based FieldTrip toolbox (Oostenveld et al., 2011). Artifacts were detected in a semiautomatic manner using visual inspection and selecting a threshold for each subject (Horschig et al., 2015). Trials containing ocular or muscle-artifacts were excluded from analysis (on average 31%). This was done for each subject individually since the absolute values of power differ largely between subjects and hence a general cut-off value would not have been useful. Datasets with too many lost trials (more than 1 SD above the average amount of lost trials) were excluded from the EEG analysis (5 data sets were excluded). This lead to 32 included data sets for EEG analysis (44 recorded subjects, –3 drop outs, –4 data sets with technical problems, –5 data sets with too few trials for statistical analysis), leading to 32 included data sets for the EEG analysis. The remaining EEG data looked as clean as in a sitting position.

Sensor level time-frequency analysis of power was performed using a sliding time-window of 500 ms and Hanning taper for the lower frequencies (2–30 Hz). This resulted in, e.g., a 5 cycle window for 10 Hz. For the higher frequencies (30–120 Hz) we used a 400 ms sliding time window and a multi-taper approach [discrete prolate spheroidal sequences (DPSS), Percival and Walden, 1993]. This resulted in, e.g., a 20 cycle window for 50 Hz. To aim for a spectral smoothing of 10 Hz we used 7 DPSS tapers. The difference in parameters between low and high frequencies is due to the different spectral smoothing requirements of, e.g., the alpha and gamma band oscillations (see Ferrante et al., 2022).

#### Electroencephalography statistical analyses

Statistical analysis was performed on the whole brain sensor level, i.e., including all sensors and frequencies. For each subject, trials were averaged per picture condition (pleasant, neutral-in-pleasant-block, unpleasant, and neutral-in-unpleasant-block) from −1 to 2 s around stimulus onset. To assess whether sensor level power was significantly different between conditions, a cluster based, non-parametric permutation test was used within subjects (Maris and Oostenveld, 2007). This test clusters neighboring sensors and time-frequency points which show the same effect and thereby controls for multiple comparisons. For each grid point (space by time by frequency point) a dependent samples *F*-test (four picture categories) was computed. For each point the *F*-value was calculated and clusters on the basis of spatial adjacency were formed where these values exceeded the critical threshold of *p* = 0.05 (uncorrected). The sum of the *F*-values within a cluster was used as cluster-level statistic and the cluster with the maximum sum was used for the test statistic. To create a reference distribution to obtain a maximum cluster *F*-value for statistical comparison with the present data, 1,000 permutations were performed in which the data of the four conditions was randomized and the test statistic recalculated. The same principle was applied for *t*-tests for block-comparison and *post-hoc* analyses. The test was first conducted on the inter trial interval (1 s before picture onset) between the two block types (pleasant vs. unpleasant block) to test for state effects. After this test, the pre-stimulus interval (−800 to −200 ms; for each of the four picture conditions separately) was used as baseline interval like in classic EEG designs (Aftanas et al., 2002; De Cesarei and Codispoti, 2011; Haegens et al., 2012) and the cluster-based permutation test was conducted for the full window of −1000 to 2000 ms around stimulus to test for stimulus induced effects.

#### Stabilometric platform

Offline, the data were low-pass filtered at 10 Hz. Trials with a z-score >4 (scores computed for each participant over all trials) were regarded as outliers and removed from analyses (on average 4%). The standard deviation of the movement in anterior-posterior direction (SD-AP), medio-lateral direction (SD-ML), and the sway path length (SPL) were calculated per trial over the stimulus period (0–2 s), normalized to the baseline period (−1–0 s), z-scored, and then averaged per picture category (Stins and Beek, 2007; Hagenaars et al., 2012). Statistical analysis was conducted in SPSS 23 (SPSS, IBM Corp, 2015).

## Results

Electroencephalography data were acquired during a passive viewing task in which subjects watched blocks of *either* unpleasant and neutral *or* pleasant and neutral pictures while standing on a stabilometric platform.

### Electroencephalography stimulus induced rather than state effects

**FIGURE 2:**
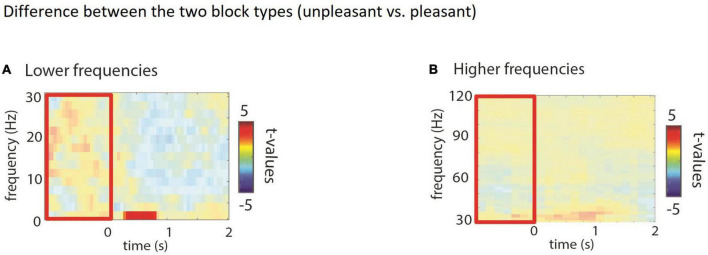
**(A,B)** Comparing the inter trial interval (red box). Time-frequency representations of power when comparing blocks (*t*-statistics), masked for significance. No significant difference in power neither in the low nor the high frequencies during the pre-stimulus interval was observed.

We first tested for stimulus induced vs. state/block effects by contrasting the inter trial interval (1 s prior to picture onset) between the two block types. The *t*-test over this period yielded no significant differences between the pleasant and the unpleasant block, neither in the lower (*p* = 0.28; Figure 2A) nor the higher frequencies (*p* = 0.53; Figure 2B). From this part of the analysis we conclude that the emotional pictures did not induce longer lasting effects on EEG power (beyond the time window of stimulus presentation).

### Electroencephalography stimulus induced effects: The alpha band

**FIGURE 3:**
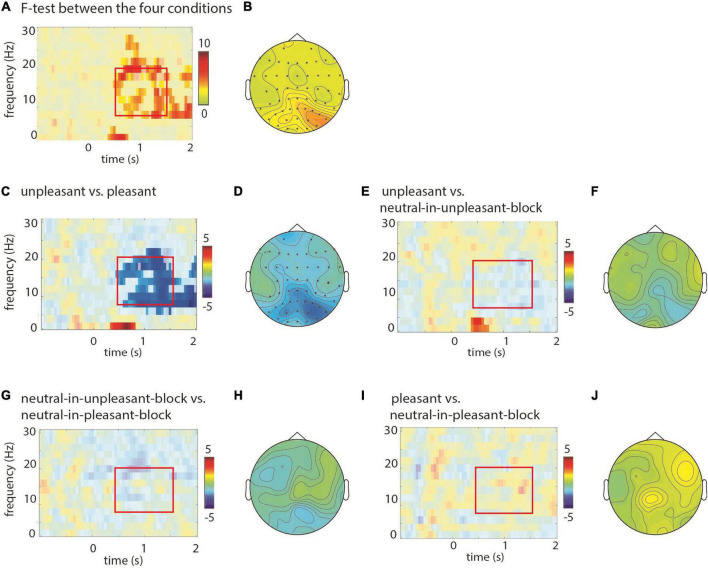
Power modulations at lower frequencies with respect to valence of the pictures. Time-frequency representations of power when comparing conditions (*F/t*-statistics), masked for significance, at sensor PO3. Topographical representations are based on averaged data for the latency 0.5–1.5 s and a frequency range from 8 to 20 Hz. Significant sensors are displayed with an asterisk in the topographical representation. **(A)** The *F*-test between the four conditions revealed that alpha and beta power is modulated in a condition specific manner (*p* < 0.05; controlled for multiple comparisons). **(B)** Topographical representation for the effect shown in panel **(A)** revealed posterior modulation. **(C,D)** A stronger decrease in alpha and beta power was observed posteriorly for unpleasant compared to pleasant pictures. **(E,F)** Apart from the evoked activity (the low frequencies <5 Hz) there was no significant difference between the unpleasant and neutral-in-unpleasant-block pictures. **(G,H)** There was no difference when comparing neutral pictures from the unpleasant and pleasant blocks, **(I,J)** neither for pictures of the pleasant vs. neutral-in-unpleasant-block.

Next we investigated the modulation in the lower frequencies (2–30 Hz) by the different picture categories. As seen in Figure 3A, the cluster based permutation *F*-test revealed robust differences in the in alpha and beta band (8–20 Hz) with respect to the four conditions over posterior sensors (Figure 3B; *p* < 0.001). Follow-up *post-hoc* pair wise comparisons (*t*-tests) showed a stronger alpha-beta power decrease for the unpleasant compared to the pleasant pictures (Figure 3C; *p* < 0.01) over all sensors with the strongest effect over posterior sensors (Figure 3D). The unpleasant compared to the neutral-in-unpleasant-block pictures showed a significant difference (*p* = 0.01), but when inspecting the according figure (Figure 3E) it becomes apparent that this contrast is clearly not dominated by alpha band activity. When excluding the evoked part (i.e., frequencies up to 5 Hz, see Karakaş et al., 2000; Herrmann et al., 2014) from the test, the difference was no longer significant (*p* = 0.17; Figures 3E,F). The contrasts between the pleasant and neutral-in-pleasant-block (*p* = 0.16; Figures 3I,J) and between the neutral pictures of the two block types did not yield significant differences (*p* = 0.07; Figures 3G,H). In short, our findings demonstrate that the suppression of the alpha and beta band activity increased with unpleasant pictures and this effect seems to be most pronounced between 0.5 and 1.5 s after stimulus onset.

### Electroencephalography stimulus induced effects: The gamma band

**FIGURE 4:**
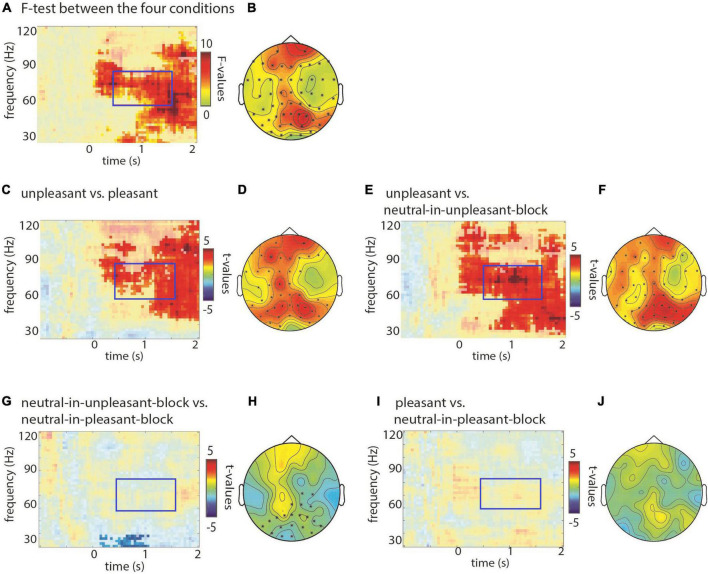
Modulations at higher frequencies with respect to valence of the pictures. Time-frequency representations of power when comparing conditions (*F/t*-statistics), masked for significance, at sensor Pz. Topographical representations are based on averaged data for the latency 0.5–1.5 s and a frequency range from 60 to 80 Hz. Significant sensors are displayed with an asterisk in the topographical representation. **(A)** The *F*-test revealed that gamma power was differently modulated by conditions (*p* < 0.05; controlled for multiple comparison). **(B)** Topographical representation for the effect shown in panel **(A)** revealed posterior and anterior modulation. **(C,D)** A stronger increase in gamma power was observed posteriorly and anteriorly for unpleasant compared to pleasant pictures. **(E,F)** Similarly, for unpleasant compared to neutral-in-unpleasant-block a strong increase in gamma power was observed, mainly over posterior sensors. **(G,H)** When comparing neutral pictures of the unpleasant and pleasant blocks, a gamma decrease at the lower frequencies around 30–40 Hz for neutral-in-unpleasant block became apparent. **(I,J)** No significant difference for pictures of the pleasant vs. neutral-in-pleasant-block was found.

Next, we set out to investigate the modulation in the higher frequencies (30–120 Hz), applying the same cluster based *F/t*-test procedure as to the lower frequencies. We found a main effect in the gamma band (60–80 Hz; at latency >1 s for an even broader band) when comparing the four picture categories using an *F*-test (*p* < 0.001; Figures 4A,B). A *post-hoc t*-test revealed a significant difference between the unpleasant and the pleasant pictures (*p* < 0.01; Figures 4C,D) with stronger gamma band activity for the unpleasant pictures. Also when compared to the neutral pictures in the unpleasant block, the unpleasant pictures elicited significantly more gamma activity (*p* < 0.01; Figures 4E,F). The contrast between the pleasant and neutral-in-pleasant-block did not yield any significant differences (*p* = 1; Figures 4I,J). The contrast between the neutral pictures in the unpleasant and the pleasant block did not yield any significant difference in the higher gamma band, but did show a difference in the 30–40 Hz band with gamma power being stronger for the neutral-in-pleasant-block pictures (*p* = 0.04; see Figures 4G,H).

In short, our findings demonstrate a stimulus induced increase in the higher gamma band activity for unpleasant pictures, while the lower gamma band activity (30–40 Hz) showed a block effect for neutral pictures (unpleasant vs. pleasant block) over temporo-posterior sites. This effect seems to be most pronounced between 0 and 2.0 s.

### Body sway effects: Stabilometric data

A repeated measures MANOVA with the factor emotion (four levels: pleasant, unpleasant, neutral-in-pleasant-block, neutral-in-unpleasant-block) was carried out on the three dependent variables (SD-ML, SD-AP, and SPL) yielding no significant effect [Wilks’ Lambda = 0.64, *F*(9,25) = 1.55, *p* = 0.19]. Means (SDs) are listed in Table 1.

**TABLE 1:** Body sway measures per condition.

	Unpleasant		Neutral-in-unpleasant-block		Pleasant		Neutral-in-pleasant-block	
	M	*SD*	M	*SD*	M	*SD*	M	*SD*
SD-ML	0.002	0.15	0.015	0.11	−0.001	0.12	0.021	0.15
SD-AP	0.016	0.13	−0.034	0.13	0.001	0.13	0.017	0.14
Sway path length	−0.022	0.11	0.007	0.13	−0.013	0.14	0.027	0.10

Note that these values are normalized (z-scored) per participant. *M, mean; SD, standard deviation.

### Correlations between electroencephalography and stabilometric data

In order to investigate the link between freezing and attention, we correlated the stabilometric (body sway) and EEG measures. To this end, EEG alpha power was averaged in the 0.5–1.5 s time window, over the parieto-occipital channels in the 8–18 Hz band (where we observed the strongest effect between conditions). We also averaged the gamma power (60–80 Hz) in the same time window and over the same sensors. These values were then log transformed. The body sway data were averaged over the full stimulus presentation window from 0 to 2 s. The resulting values were then z-scored and averaged per subject per condition.

A canonical correlation analysis (Sherry and Henson, 2005) evaluating the multivariate shared relationship between the EEG measures (alpha and gamma band activity) with those of the body sway measures (SD-AP, SD-ML, and SPL) for the difference scores between the conditions (unpleasant–pleasant, unpleasant–neutral-in-unpleasant-block, pleasant–neutral-in-pleasant-block, and neutral-in-unpleasant-block–neutral-in-pleasant-block) was set up. The canonical correlation did not yield any significant results [*F*(5,70.88) = 1.16, *p* = 0.25 for the strongest correlation].

## Discussion

In the current study we observed that pictures of unpleasant valence elicited a stimulus induced decrease in alpha/beta power (around 8–18 Hz) over posterior sensors and an increase in gamma power (around 60–80 Hz) over posterior and anterior sensors when compared to pleasant pictures, as well as a gamma increase when compared to neutral pictures within the unpleasant block. Pleasant compared to neutral pictures did not show a difference in either alpha or gamma power. Furthermore, we explored whether these attention effects pertain to a state effect over a whole block or are related to single pictures (i.e., stimulus induced). As the ITI did not show a difference between the pleasant and unpleasant blocks, and furthermore the contrast between the *neutral* pictures from the two block types did not show a difference (except for a relative decrease in power around 30 Hz for neutral pictures from the unpleasant block), we conclude that the effects of emotional attention are stimulus induced rather than state effects.

Finally, we tested whether this emotional modulation of brain responses was linked to decreased body sway as a measure of freezing. The body sway data showed neither a main effect of emotion condition nor any correlation with alpha or gamma power. In light of the results from previous research on selective attention (Fries et al., 2001; Jensen and Mazaheri, 2010; Foxe and Snyder, 2011; Haegens et al., 2012) we interpret our findings of alpha/beta power decreases and gamma increases as enhanced attention toward unpleasant stimuli. These findings will be discussed in detail below.

### Alpha power

#### Attention toward unpleasant pictures

The decrease in alpha power toward unpleasant compared to pleasant pictures found in the present study is generally in line with previous research (Keil et al., 2001; Popov et al., 2012). However, there are a few distinctions between our study and previous studies. Popov et al. (2012) found a significant alpha decrease for unpleasant compared to neutral pictures in a passive viewing task. Our data did not show this effect in the alpha band, yet, we did find increased gamma power for unpleasant compared to neutral pictures, pointing in the direction of an attention increase for unpleasant compared to neutral pictures. The difference in the alpha band finding may be due to differences in study designs.

#### An effect related to events

While balance board studies involving emotions mostly found effects using block designs (Azevedo et al., 2005; Facchinetti et al., 2006; Hagenaars et al., 2012, 2014; Bastos et al., 2016) our study showed emotion effects related to events on a short time-scale (stimulus induced), at least in the EEG (and no effects in the balance board). The absence of a block effect may be due to the semi-blocked design of our study, which was a hybrid between a classic (blocked) balance board design and a classic (fully randomized) EEG design. Stins and Beek (2007) already found that a passive viewing task with a fully randomized design induces too weak effects of emotion on body sway to be measured by the balance board. This also seems to be true for our semi-blocked design.

Our EEG results suggest that even in a semi-blocked design (as opposed to a fully randomized design), the increases in attention to unpleasant pictures are stimulus induced rather than state (i.e., block) effects, which is in line with previous research (Schupp et al., 2013). Future studies investigating concurring effects of body sway and brain activity may want to take a step closer toward the classic balance board designs, for example by leaving out ITIs between stimuli while keeping a semi-blocked design, or by keeping the ITIs but using a fully blocked design in terms of valence, in order to be able to find freezing-like effects in body sway and potentially link them to attentional effects of emotion.

In principle, the effects of the pictures can represent a short lasting effect or carry on for longer. Our data of the ITI seems to speak to the shorter lasting effect. Yet, the significant difference between neutral pictures from unpleasant vs. pleasant blocks in the gamma band may indicate a state-related oscillatory brain response as well. It may be the case that short lasting effects play out in the alpha band while longer lasting effects play out in the gamma band. This may pose an interesting avenue for future research.

#### A central-posterior effect

While the effects of emotion on alpha or gamma power in other studies were often either hemifield, hemisphere or site specific, or including interactions between these factors (Mueller et al., 1999; Keil et al., 2001; Balconi et al., 2009), we found a central posterior alpha/beta (and frontal gamma) effect. While previous studies mostly analyzed pre-defined sites, we used the cluster-based permutation test over the whole brain. This difference in analysis method may have led to the difference of results in terms of the location of the emotion effects; however, the differences might also be explained by the details of the experiments not being the same.

#### Role of arousal

Aftanas et al. (2002) and De Cesarei and Codispoti (2011) found more alpha suppression for arousing pictures than for non-arousing pictures, independent of valence. In our design we did not see arousal modulating alpha/beta suppression, i.e., *p* = 0.09 for the high arousing emotional pictures (pleasant and unpleasant taken together) compared to the low arousing neutral pictures (of both blocks taken together). The mean arousal rating of our emotional pictures, based on the normative ratings of the IAPS database, was 5.55 (*SD* = 0.97) and of the neutral pictures 3.39 (*SD* = 0.70). The difference between our result and the one of De Cesarei and Codispoti (2011) could be explained by a difference in stimuli, as they used also pictures of other origin than the IAPS which might have been even more arousing than the ones we used from the IAPS database. To better address this issue, future studies would ideally include individual arousal ratings to be able to control for possible differences in arousal perception.

Uusberg et al. (2013) found less alpha power decrease over dorsal and central sites for emotional and in particular unpleasant (aversive) pictures, i.e., the opposite of De Cesarei and Codispoti (2011) and the present study. Uusberg et al. (2013) reason that their results are due to the difference in task: In their study the subjects had to make evaluations (affective and non-affective), whereas in the study by De Cesarei and Codispoti (2011) the instruction was to just perceive the picture (passive viewing task). Since we also used a passive viewing task, our results support this interpretation. It seems that conducting a task (e.g., evaluating aspects of a picture) leads to guarding the attention from distracting input like, e.g., too strong emotional input. Unpleasant pictures seem to require more attentional resources to ignore, than pleasant and neutral ones in the case where they interfere with task goals. This is in line with the findings by Haegens et al. (2012) who showed that stronger distracting input needs more alpha power in order to attenuate the distracting input than weak distracting input. This is in line with attention models differentiating between top-down (e.g., instructed) and bottom-up (stimulus-driven) attention (see e.g., Compton, 2003).

#### Beta band

Some of the activation differences we see may stretch into the beta band (ca. 13–23 Hz). Other studies have found results in this range with similar images, namely, Güntekin and Tülay (2014) who showed higher beta power in response to unpleasant pictures compared to pleasant and neutral pictures and Güntekin and Başar (2010) who indicated greater beta responses for negative images compared to positive images. Our results are thus in line with these findings.

#### Lower frequencies

We did find an effect at lower frequencies (<5 Hz; see Figures 2, 3). For unpleasant vs. positive this effect speaks to enhanced attention for unpleasant pictures, similar to the decrease in alpha activity in the same contrast. We also observed a small effect for unpleasant vs. neutral pictures which would indicate increased attention for the unpleasant pictures as well. Due to the small size of this cluster, we remain conservative in interpreting this finding.

### Gamma power

Our result of increased gamma power for (high arousing) unpleasant pictures compared to (low arousing) neutral pictures is in line with the literature, though often these effects are found in the 30–60 Hz range (Mueller et al., 1999; Keil et al., 2001; Aftanas et al., 2004; Balconi et al., 2009; Güntekin and Tülay, 2014) while our effects were most pronounced in the 60–90 Hz band. Our study distinguishes itself from those studies in allowing the cluster-based permutation test to identify effects up to 120 Hz rather than pre-defining and hence limiting the frequency range in the analysis, which may explain this difference in findings. Another difference in findings is that those studies found an effect of arousal: they found increased gamma toward high arousing as opposed to low arousing pictures (both valences together). Our findings in contrast show an effect of valence: the unpleasant pictures elicited enhanced gamma activity compared to neutral but also compared to (high arousing) pleasant pictures. This could possibly be linked to the difference in the frequency range in which the effects were found.

#### Comparing alpha and gamma results

Our alpha and gamma findings support each other, being in line with computational frameworks which explain that a decrease in alpha band activity would support active processing which then is reflected by increased activity in the gamma band (Jensen and Mazaheri, 2010; Jensen et al., 2014; Hyafil et al., 2015; Schalk, 2015). Taken together, our results of increased gamma and decreased alpha power suggest a clear increase of attention toward unpleasant pictures.

### Body sway

Our measure of freezing, as reflected by body sway, did not yield any significant effects of emotion in the current design; neither main effects of picture condition, nor when correlating the condition contrasts of the balance board with the brain activity in the alpha and gamma band. We strongly believe that this is due to the task setup. Compared to the classic design of balance board studies (Azevedo et al., 2005; Roelofs et al., 2010; Hagenaars et al., 2012; Noordewier et al., 2020), we undertook a number of alterations, the sum of which may have altered the participant’s automatic postural response: First, there were two unnatural constraints during the passive viewing task: (1) participants had to fixate their gaze on the fixation cross and hence suppress the natural impulse to explore the picture, and (2) they were required not to blink during the presentation of the picture (and we would remind them if they did, so participants who blinked might have felt observed). Secondly, our design may have induced a less intense emotion, since we had a shorter picture presentation time (2 s instead of 3 s), used ITIs, and used a semi-blocked design. Thirdly, the passive viewing task took longer (20 min rather than 3–5 min) and thus far, it is not known what effect prolonged standing has on body sway. Fourth, wearing the EEG cap (with cables attached to the amplifier) may have affected their posture, though we asked whether they felt constrained by the cap and most subjects reported that they did not. Yet, it would be interesting to overcome these issues and be able to link attention effects in brain activity to freezing in order to disclose whether freezing is indeed a state of enhanced attention, as the animal models posit (Lang et al., 1997). In future studies, we recommend to keep the design closer to classic balance board designs, e.g., use a fully blocked design (perhaps not even with an inter trial-interval between stimuli within a block) and use a pre-experimental period, or a short interval between blocks, as baseline for the EEG.

## Conclusion

Our findings reveal an attentional increase toward unpleasant pictures as identified by a robust increase of gamma activity for unpleasant as compared to pleasant and neutral pictures, as well as alpha suppression for unpleasant compared to neutral stimuli, speaking to increased attention toward unpleasant pictures given the functional role of these oscillatory activities. In addition, the lack of a difference in brain oscillatory activity during the inter trial interval between pictures and between the neutral pictures presented within the unpleasant and pleasant blocks suggest that attention to emotional pictures is directed in a stimulus induced manner rather than reflecting a state that is maintained throughout a block. In our semi-blocked design we observed no emotion related changes in body sway. In future research, an adapted design may allow researchers to combine measures of brain activity and body sway, in order to elucidate the neural correlates of freezing and the predicted relation between freezing and enhanced attention.

## Data availability statement

The datasets presented in this article are not readily available because the data are archived at the Donders Institute, Nijmegen, Netherlands. The involved authors have moved away from this university. Theoretically the data can be handed out, but one would need to get to Nijmegen and find a person who can hand them out. Requests to access the datasets should be directed to admin@donders.ru.nl.

## Ethics statement

The studies involving human participants were reviewed and approved by Ethische Commissie Gedragswetenschappelijk onderzoek, ECG, of the Behavioural Science Institute at the Radboud University Nijmegen, Netherlands. The patients/participants provided their written informed consent to participate in this study.

## Author contributions

LL was responsible for the data collection and drafted the manuscript. LL performed the EEG data analysis and interpretation under the supervision of OJ with input by JH. LL performed the body sway data analysis and interpretation under supervision of OJ, KR, and MH. All authors contributed to the study concept and design, provided revisions, and approved the final version of the manuscript prior to submission.

## Appendix

### Numbers of the International Affective Picture System pictures.

**Table:**

**Neutral**												
2215	1675	7004	7034	9070	7175	5510	7205	2880	7040	7031	2280	7493
2191	2305	1670	7036	7490	2489	7100	7080	5395	7224	7033	2635	7000
7504	7236	2595	2211	2410	9210	7011	3550.2	2038	7001	7060	7090	2480
2870	7041	2273	7300	2493	2484	2411	2840	7030	1903	2026	7035	2441
5471	2002	7512	7640	2383	7500	5740	5040	5520	4605	8325	2382	7487
7950	7081	2597	7161	7026	2495	7057	2780	2381	7025	2221	7285	7513
7595	2749	7550	2720	5731	7187	2102	7179	7002	8121	7497	7044	2210
2385	2020	2272	7050	2830	2104	7190	2514	2200	7061	2390	7037	2397
2487	2101	7290	8312	7010	1616	7705	7009	2230	7217	2351	2393	7006
2396	2579	7235	7546	9700	9260	9468	2512	7207	2357	2575	2518	7150
2372	8010	2440	2377	2506	7491	6150	7185	7062	2890	5534	1935	2214
2235	2485	2499	2107	7140	2190	1313	2570	7211	1350	2516	7020	7506
**Pleasant**												
8041	4641	5600	8161	5210	1750	8186	8350	7330	8192	4626	8620	8400
1460	4599	2310	8190	5825	1731	5760	2045	4623	2071	5700	8210	1440
8461	8032	5621	8470	2216	1463	7200	2550	1811	5982	1999	8490	1920
2158	8120	2540	8370	5829	2170	8300	8460	2154	1722	8034	8540	8040
2332	5833	5480	8021	2306	2340	7508	1610	5631	5594	5200	2035	2391
8200	8090	1441	2311	1710	2373	8380	2208	8465	5780	2398	5910	5010
**Unpleasant**												
9419	3100	2375.1	9006	3180	3069	9904	9410	3150	3010	3102	6540	9428
9414	3053	9040	3130	9000	3181	9903	9340	9920	6212	9322	9570	3131
9901	3225	3266	9830	3016	3051	3195	9342	9940	3063	2703	3060	2900.1
3160	7380	9183	9560	9325	9520	6230	2352.2	9254	3400	3191	3080	9185
9184	3000	3110	9140	3015	3030	9181	9413	3061	9810	6311	9331	6838
3062	2717	2799	9405	3140	2205	9290	2095	3170	3261	9611	3064	9250
